# Comprehensive genomic profiling aids in understanding the lesion origins of a patient with six synchronous invasive lung adenocarcinomas: a case study

**DOI:** 10.1186/s12890-020-1119-9

**Published:** 2020-04-03

**Authors:** Yang Song, Ziqi Jia, Pancheng Wu, Weiwei Wang, Qiuxiang Ou, Hua Bao, Man Yu, Xue Wu, Peng Liu, Naixin Liang, Shuyang Zhang, Shanqing Li

**Affiliations:** 1 Department of Thoracic Surgery, Peking Union Medical College Hospital, Chinese Academy of Medical Sciences, Beijing, 100730 China; 2 Peking Union Medical College, Eight-Year MD Program, Chinese Academy of Medical Sciences, Beijing, 100730 China; 3 Peking Union Medical College Hospital, Chinese Academy of Medical Sciences, Beijing, 100730 China; 4 Translational Medicine Research Institute, Geneseeq Technology Inc., Toronto, Canada; 5 Medical Research Center, Central Laboratory, Peking Union Medical College Hospital, Chinese Academy of Medical Sciences, Beijing, 100730 China; 6 Department of Cardiology, Peking Union Medical College Hospital, Peking Union Medical College & Chinese Academy of Medical Sciences, Beijing, China

**Keywords:** Synchronous multiple primary lung cancer, sMPLC, Prognosis, Whole-exome sequencing

## Abstract

**Background:**

Synchronous multiple primary lung cancers (sMPLC) are rare forms of lung cancer, and their diagnosis remains as a significant challenge. Distinguishing sMPLC from advanced disease is important as their prognoses and therapeutic management vary dramatically.

**Case presentation:**

The patient was a 56-year-old Chinese male who exhibited six synchronous invasive adenocarcinomas at diagnosis [T2(6)N0M0], and who achieved durable clinical benefit under adjuvant chemotherapy for 41 months following wedge resection and lobectomy. Whole-exome sequencing revealed that two lesions (L4 and L6) in the left upper lobe of the patient’s lung shared 28 nonsynonymous mutations; thus, suggesting that the lesions may have arisen from a common ancestor at the early stages of tumorigenesis, and spread into distinct histologic subtypes. Moreover, while L5 was in the same lobe as L4 and L6, it represented a distinct lineage as it did not share any mutations with other lesions. Notably, the *BRAF* V600E oncogenic mutation was exclusive to L5. In addition, the *KRAS* G12C mutation was identified in three lesions (L1-L3) located in the right lung, which may have resulted from convergent evolution.

**Conclusion:**

We report a patient with six synchronous invasive adenocarcinomas who demonstrated durable clinical benefits under adjuvant chemotherapy following surgical treatment. While cancer staging is one of the many challenges associated with sMPLC, the data generated through next-generation sequencing can provide information on lesion origins, and thus, advance the era of precision medicine.

## Background

An increasing number of lung cancers (~ 15% of surgical patients) exhibit two or more malignant pulmonary lesions [1, 2]; however, synchronous multiple primary lung cancer (sMPLC) remains a rare form of lung cancer [3]. Distinguishing sMPLC from advanced disease is clinically important as the prognosis and treatment vary between the two forms of disease, and an aggressive surgical approach to sMPLC may result in rates of survival comparable to single lung cancers of similar stage [4, 5]. Thus, in the 8th edition of the Tumour, Node and Metastasis (TNM) Classification of Lung Cancer [6, 7], the International Association for the Study of Lung Cancer (IASLC) proposed that a unique staging strategy be applied to multiple ground glass opacities (GGOs) suspected of being sMPLC. While the current diagnosis of sMPLC uses the criteria defined by Martini and Melamed [8], molecular evaluation of multiple lesions has become increasingly valued for conceptually understanding the nature of such lesions, as well as the lineages (clonality) between lesions [9, 10]. The precise interpretation of the clonal origin of sMPLCs will facilitate the rationalization of treatments for sMPLC patients and improve their prognosis.

## Case presentation

A 56-year-old Chinese male patient was admitted to the Hospital following the accidental discovery of GGOs in his lung during a routine physical examination. The patient was a heavy smoker for 30 years. Routine laboratory workups and the levels of serum tumor markers including carcinoembryonic antigen (CEA) were normal. Microbiological blood tests were negative, and no abnormal cells were detected during the sputum cytology test. Computed tomography (CT) scans revealed bilateral and ill-defined GGOs, including three lesions (L4-L6) in the left upper lobe (LUL), and three additional lesions, L1-L3, in the right upper lobe (RUL), right middle lobe (RML), and right lower lobe (RLL), respectively. A Corona radiata sign was observed in L1, which was in the apical segment of the RUL with the convergence of the supplying blood vessels. Cavitation was present in L3 and there was no associated lymphadenopathy in the mediastinum.

**Fig. 1:**
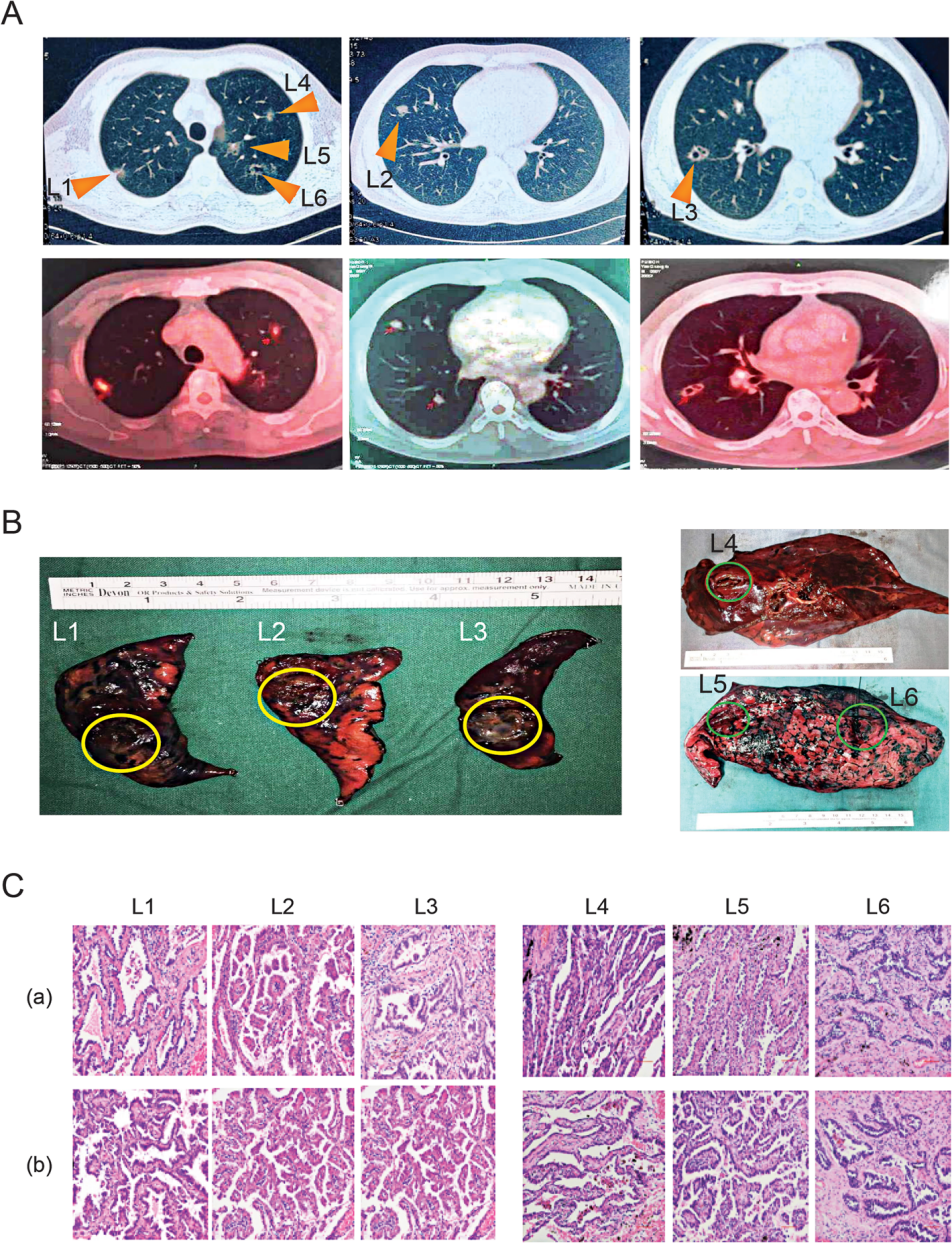
Chest radiograph and clinicopathologic details of the lesions. **A** Upper panel: Chest computed tomography scan: L1, L2 and L3 were in the right upper lobe (RUL), right middle lobe (RML) and right lower lobe (RLL), respectively. L4, L5 and L6 were all observed in the left upper lobe (LUL). No hilar or mediastinal lymphadenopathy was observed. Bottom panel: The corresponding positron emission tomography (PET-CT) scans revealed intense 18F-fluorodeoxyglucose (FDG) avidity. **B** Tumor lesion images. **C** Microscopic images of hematoxylin and eosin-stained sections representing the predominant histologic subtypes. Two representative areas are shown in (a) and (b). The corresponding histologic subtypes are described in Table 1

A positron emission tomography (PET) scan revealed increased 18F-fluorodeoxyglucose (FDG) avidity in these multifocal lesions. The size of the lesions ranged from 0.7 cm to 2.4 cm, with maximum standardized uptake values (SUV_max_) ranging from 1.3 to 3.8. No distant metastases were detected (Fig. 1).

Each lesion was larger than 6 mm, and according to the 2017 Fleischner Society guidelines [11], all six lesions were suspected of being adenocarcinomas. Considering the National Comprehensive Cancer Network guidelines (NCCN®, 2019.V2), radical resection procedures were recommended as follows: L4-L6 subject to complete resection by a preferred anatomic left upper lobectomy, while L1-L3 were appropriate for wedge resections to preserve the patient’s lung function. Therefore, the patient underwent video-assisted thoracoscopic (VATS) right lobe wedge resections, followed by VATS LUL lobectomy 1 month later. Associated mediastinal lymph node dissection, as well as systematic lymph node sampling was also performed (thoracic lymph nodes dissected were stations 9R, 7, 10R, 11R, 2R, 4R for the right wedge resections, and stations 9 L, 7, 4 L, 5, 10 L, 11 L, 12 L for the LUL lobectomy). Pathological analyses of the surgically resected specimens revealed the six lesions as invasive pulmonary adenocarcinomas. The resection margins of the resected lung tissues were clean. L1 was reported to invade the visceral pleura (PL1), but no bronchus involvement was observed in any lesions. Additionally, no metastases were detected in any of dissected hilar or mediastinal lymph nodes.

The immunohistochemistry (IHC) data revealed that the lesions exhibited different histological subtypes, including 50% papillary + 50% acinar in L1, L3 and L5; 90% papillary in L2; 90% lepidic in L4; and 90% acinar in L6. The detailed clinicopathological characteristics of the lesions are summarized in Table 1.

**Table 1:** Patient clinicopathological characteristics

Sex	Age (years)	Smoking status	Presentation	Tumor	Location	Size (mm)	Radiological feature	Histology	Subtype					Nonsynonymous mutation# (WES)
									Papillary	Acinar	Lepidic	Micropapillary	Solid	
Male	56	Heavy smoker (>30y)	Synchronous	L1	RUL	20 × 16 × 12	GGO	ADC	50%	50%	–	–	–	203
				L2	RML	19 × 16 × 10	GGO	ADC	90%	–	–	–	–	213
				L3	RLL	24x17x11	GGO	ADC	50%	50%				164
				L4	LUL	13 × 10 × 6	GGO	ADC	–	–	90%	–	–	185
				L5	LUL	14 × 9 × 5	GGO	ADC	50%	50%	–	–	–	123
				L6	LUL	17 × 10 × 9	GGO	ADC	–	90%	–	–	–	182

According to the Martini-Melamed classification (1975), the patient was diagnosed with sMPLC [8]. The pathologic stage of L1 was T2 due to visceral pleura invasion (PL1). L3 had a maximum diameter of 24 mm and was defined as T1c, while the remaining four lesions were identified as T1b. According to the IASLC guidelines for sMPLC staging [6], the patient was determined to be pT2(6)N0M0 (T category was designated by the category of the highest T lesion, while the N and M categories were designated collectively for all lesions). Considering the high-risk factors, including visceral pleural involvement and wedge resection, and the lack of an appropriate targeted therapy, the patient received four cycles of chemotherapy with a conventional regimen of Pemetrexed (950 mg) and cisplatin (140 mg). No recurrence was observed during the 41-month follow-up period.

**Fig. 2:**
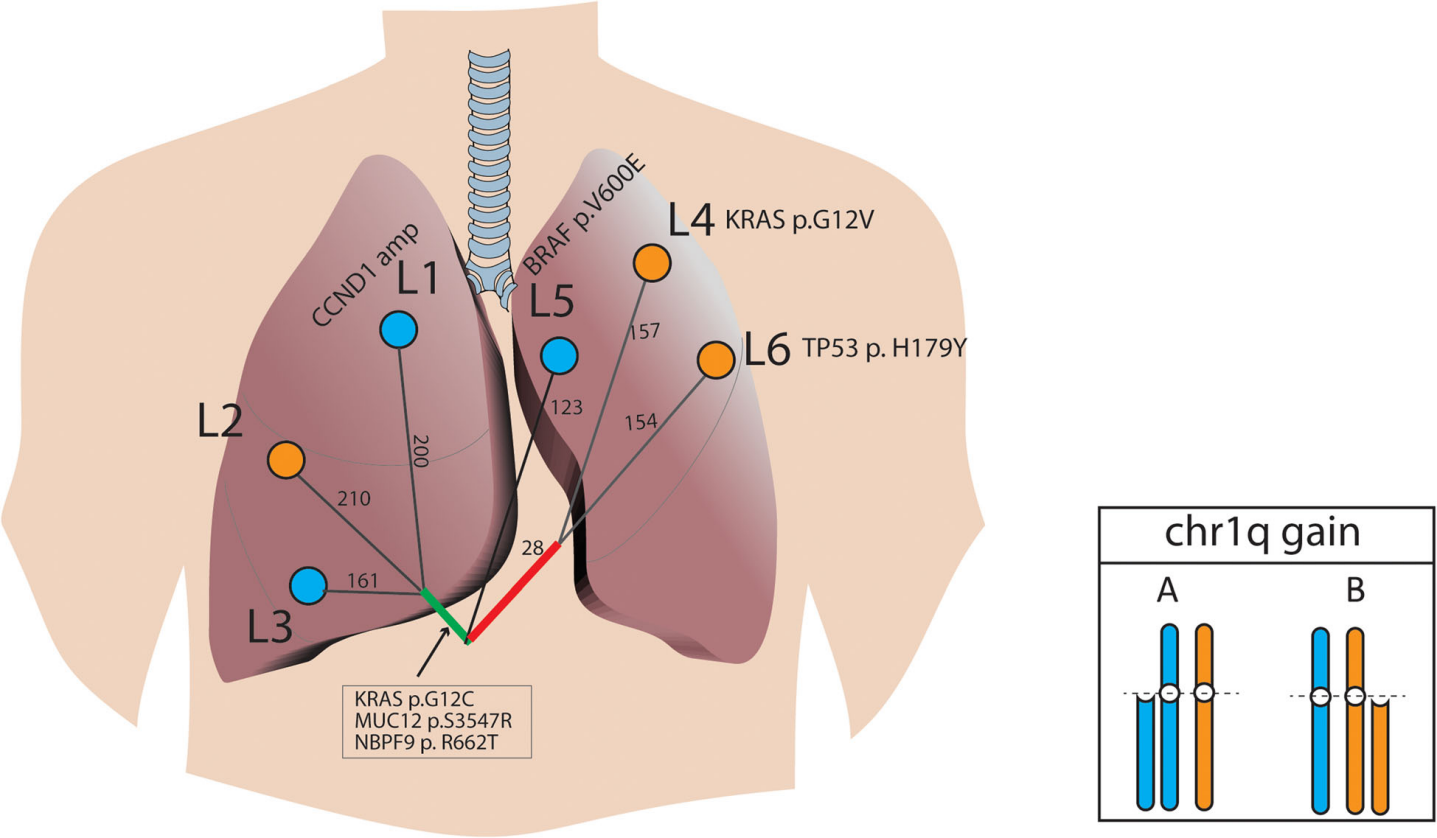
Phylogenetic analysis of six tumor lesions. Mutational profiling using whole-exome sequencing revealed that L4 and L6 shared a total of 28 nonsynonymous mutations. L1, L2, and L3 shared three nonsynonymous mutations, including the *KRAS* G12C hotspot mutation. L5 represented an independent lesion of distinct lineage. The numbers of nonsynonymous mutations detected in different lesions are indicated with the representative mutations shown. Chr1q gain was detected in all lesions. The colors indicate the chr1q gain as either paternal or maternal based on the analysis of the alternate allelic frequencies of heterozygous SNPs (refer to Figure S1 for additional details)

To investigate the molecular profiles of the different lesions, genomic DNA was extracted from formalin-fixed, paraffin-embedded tumor specimens using QIAamp DNA FFPE Tissue Kit (Qiagen), and library preparations were performed with KAPA Hyper Prep Kit (KAPA Biosystems). Target enrichment was performed using the xGen Exome Research Panel and Hybridization and Wash Reagents Kit (Integrated DNA Technology) according to the manufacturer’s protocol. Sequencing was performed on Illumina HiSeq4000 platform using PE150 sequencing chemistry (Illumina) [12]. To determine if lesions originated from the same ancestral clone, we required at least four nonsynonymous mutations (excluding driver mutations) to be shared by two independent tumors in a cohort of 126 lung adenocarcinomas at a probability of 0.1%, under the assumption that each patient’s tumor was of an independent origin [13]. Our data revealed that L4 and L6 shared a total of 28 nonsynonymous mutations, strongly suggesting that those lesions arose from a single clonal event during the early stages of tumorigenesis, and subsequently evolved into different histological subtypes (Fig. 2). The analysis of the alternate allelic frequency of heterozygous single nucleotide polymorphism (SNPs) further supported that the chr1q gain detected in L4 and L6 were likely of the same origin (paternal or maternal) (Figure S1).

Although L5 was in the same lobe as L4 and L6, it arose from an independent lineage, as no mutations were shared between it and the other lesions. Additionally, the *BRAF* V600E oncogenic mutation was exclusive to L5 (Fig. 2). Conversely, L1-L3 shared three nonsynonymous mutations, including the *KRAS* G12C driver mutation, which was more likely to have resulted from convergent evolution, rather than being derived from a common ancestral clone.

## Discussion and conclusions

sMPLC is a rare form of lung cancer, and its diagnosis remains as a significant challenge. It is critical to distinguish sMPLC from intrapulmonary metastases as the therapeutic approach and prognosis for the two conditions are markedly different. Herein, we report a 56-year-old Chinese male patient with six synchronous invasive adenocarcinomas who achieved durable clinical benefit following adjuvant chemotherapy for 41 months after surgery. This observation was consistent with previous findings that multifocal disease is a heterogenous category where the clinical outcomes were superior to those of a single nodule at similar stages [14].

Comparisons of the molecular profiles of different lesions revealed that lesions L4 and L6, located in the LUL, likely originated from a common ancestral clone at the early stages of tumorigenesis. Despite exhibiting different histological subtypes, L4 and L6 had 28 non-silent mutations in common. Given the absence of metastatic disease in local lymph nodes, it is possible that progenitor tumor cells underwent aerogenous metastasis, which is a discontinuous spread of cancer cells from the primary tumor through the airways to adjacent or distant lung parenchyma [15]. Furthermore, although L1-L3 were in different lobes of the patient’s right lung, they shared three nonsynonymous mutations, including *KRAS* G12C. Given the cutoff for the number of shared mutations required to define clonality, it is less likely that L1, L2, and L3 were derived from the same ancestral clone. Rather, they may have resulted from convergent evolution.

In summary, we reported a patient with six multifocal invasive lung adenocarcinomas. Given the patient’s clinicopathological characteristics and favorable prognosis, a diagnosis of sMPLC was established. However, the presence of shared mutations between lesions suggested that some lesions may have been derived from a common ancestor at very early stages of tumorigenesis. Moreover, we showed that comprehensive genomic profiling (i.e., whole-exome sequencing) provided a new approach to understanding sMPLC in the era of precision medicine.

## Supplementary information

**Additional file 1: Figure S1.** Heatmap of the alternate allelic frequencies of heterozygous SNPs on chromosome 1q across all lesions. The color indicates the alternate allelic frequency ranging from red to white (range: 0–100%). Lesions are clustered based on the similarities of the patterns of the alternate allelic frequency of heterozygous SNPs.

## Data Availability

All data supporting the conclusions of this case report are provided in the article.

## Abbreviations

CEA: Carcinoembryonic antigen
CT: Computed tomography
FDG: 18F-fluorodeoxyglucose
GGOs: Ground glass opacities
IASLC: International Association for the Study of Lung Cancer
IHC: Immunohistochemistry
LUL: Left upper lobe
NCCN: National Comprehensive Cancer Network guidelines
RLL: Right lower lobe
RML: Right middle lobe
RUL: Right upper lobe
PET: Positron emission tomography
sMPLC: synchronous multiple primary lung cancers
SNP: Single nucleotide polymorphism
SUV: Standardized uptake values
TNM: The tumour, node and metastasis classification
VATS: Video-assisted thoracoscopic
WES: Whole-exome sequencing
