# Supplementary figures and images for: Comprehensive genomic profiling aids in understanding the lesion origins of a patient with six synchronous invasive lung adenocarcinomas: a case study
Source: BMC Pulm Med. 2020 Apr 3;20:80. doi: 10.1186/s12890-020-1119-9 (PMC7118875; doi:10.1186/s12890-020-1119-9)

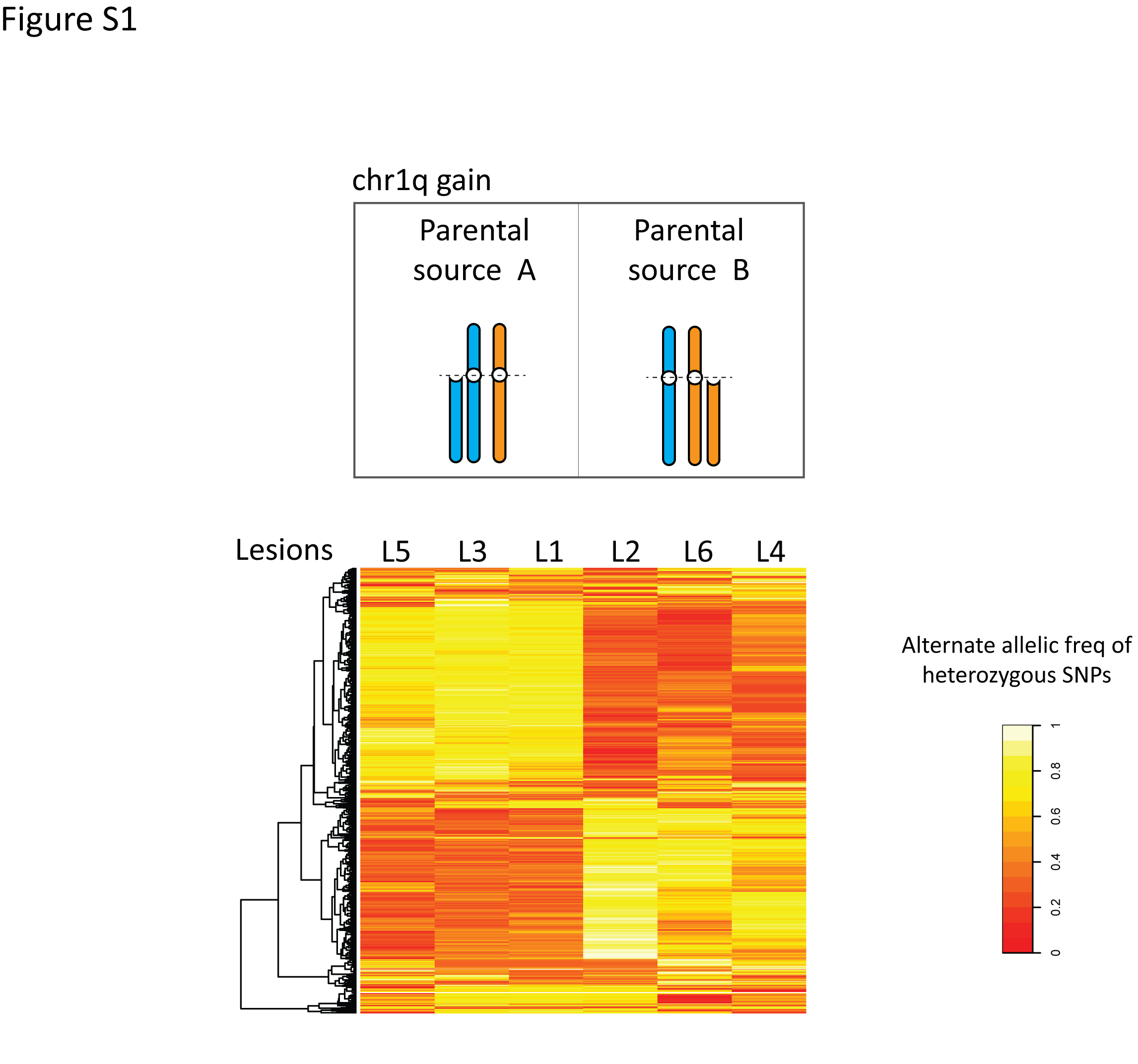

Supplement: Supplementary file 1 — Additional file 1: Figure S1. Heatmap of the alternate allelic frequencies of heterozygous SNPs on chromosome 1q across all lesions. The color indicates the alternate allelic frequency ranging from red to white (range: 0–100%). Lesions are clustered based on the similarities of the patterns of the alternate allelic frequency of heterozygous SNPs. [file 12890_2020_1119_MOESM1_ESM.tif]
